# Noninvasive monitoring of liver metastasis development via combined multispectral photoacoustic imaging and fluorescence diffuse optical tomography

**DOI:** 10.7150/ijbs.40896

**Published:** 2020-03-12

**Authors:** Jonathan Lavaud, Maxime Henry, Pascal Gayet, Arnold Fertin, Julien Vollaire, Yves Usson, Jean-Luc Coll, Véronique Josserand

**Affiliations:** 1 OPTIMAL, Small animal Imaging Platform, F-38000 Grenoble, France.; 2 INSERM U1209, CNRS UMR5309, Univ. Grenoble Alpes, Institute for Advanced Biosciences, F-38000 Grenoble, France.; 3 Fluoptics, Grenoble, France.; 4 CNRS UMR5525 ; TIMC-IMAG, Univ. Grenoble Alpes, F-38000 Grenoble, France.

**Keywords:** photoacoustic imaging, liver metastases

## Abstract

**Rationale:** *In vivo* molecular imaging in preclinical animal models is a tool of choice for understanding the pathophysiological mechanisms involved in cancer development and for conducting drug development research. Moreover, combining several imaging modalities can provide multifaceted, complementary and cross-validated information. Photoacoustic imaging (PAI) is a promising imaging modality that can reflect blood vasculature and tissue oxygenation as well as detect exogenous molecules, but one shortcoming of PAI is a lack of organic photoacoustic contrast agents capable of providing tumor contrast.

**Methods:** In the present study, we designed an animal model of liver metastases from colon cancer and monitored metastasis development by *in vivo* bioluminescence and X-ray microcomputed tomography. Contrast-agent-free PAI was used to detect the respective amounts of oxy- and deoxyhemoglobin and, thus, liver tissue oxygenation. two contrast agents, Angiostamp800 and indocyanin green (ICG), respectively with and without tumor targeting specificity, were then evaluated for their dual fluorescence and photoacoustic detectability and were then used for combined PAI and fluorescence diffuse optical tomography (fDOT) at various disease development stages.

**Findings:** Contrast-agent-free PAI reflected tumor angiogenesis and gradual hypoxia during metastasis development. Multispectral PAI enabled noninvasive real-time monitoring of ICG blood pharmacokinetics, which demonstrated tumor-related liver dysfunction. Both PAI and fluorescence ICG signals were clearly modified in metastasis-bearing livers but did not allow for differentiation between different disease stages. In contrast, there was a significant improvement achieved by using the tumor-specific marker Angiostamp800, which provided gradually increasing PAI and fDOT signals during metastasis development.

**Conclusion:** We demonstrated for the first time the value of using Angiostamp800 as a bimodal tumor-targeting contrast agent for combined PAI and fluorescence imaging of liver metastasis progression *in vivo.*

## Introduction

*In vivo* molecular imaging in preclinical animal models is a tool of choice for the fundamental research and understanding of the pathophysiological mechanisms involved in cancer development and metastasis. Molecular imaging has also become a key player in drug development research, especially for the *in vivo* evaluation of new anticancer therapeutic strategies aimed at improving cancer therapy and circumventing resistance to conventional radio- or chemotherapies. This line of research requires the establishment of relevant animal models that consistently reproduce the biological pattern of human pathology and the ability to precisely characterize their tumor development in order to apprehend the impact of new therapeutic attempts.

At the beginning of the 2000s, *in vivo* fluorescence imaging stood out in the preclinical imaging field due to significant technological advances in light detection techniques and a better understanding of the interactions of light with biological tissues 1. Further technological advancements using laser light sources and a highly sensitive camera combined with mathematical modeling of light propagation in tissues have allowed fluorescence diffuse optical tomography (fDOT) to be utilized to perform 3D reconstruction of fluorescence signal distribution within a mouse body and measure fluorophore concentrations *in vivo* in several anatomical volumes of interest, including deep organs 2-5. Nevertheless, these advances could be particularly useful because their development was concomitant with the development of biocompatible imaging tracers aimed at generating tumor contrast, based on either nonspecific accumulation due to the enhanced permeability and retention (EPR) effect 6 or specific recognition of cancer biomarkers 7 that may have an application for diagnostic, prognostic and treatment response monitoring.

Recently, photoacoustic imaging (PAI) has emerged as a new imaging technology that combines the most compelling features of optical imaging and ultrasound, providing both high optical contrast and high ultrasound resolution at depth in living organisms 8. PAI offers great promise for noninvasive exploration of biological tissues, leveraging differences in the optical absorption of underlying tissue components such as hemoglobin, lipids, melanin, collagen and water. In particular, oxy- and deoxyhemoglobin are endogenous absorbers exhibiting specific photoacoustic signals that reflect the blood vasculature as well as tissue oxygenation or hypoxia 9. PAI can also detect exogenous molecules and thus is inherently capable of being used for molecular imaging 10. Although a certain number of inorganic contrast agents that generate photoacoustic signals have already been described 10, there remains a real need for fully biocompatible organic molecules that could be used for the *in vivo* PAI of cancer.

In the present study, Angiostamp800 is introduced for the first time as a new photoacoustic organic contrast agent, whose tumor-targeting specificity can be used for the noninvasive monitoring of liver metastasis progression *in vivo*. Moreover, Angiostamp800 is shown to display bimodal properties enabling one to first perform PAI in combination with fluorescence imaging and thus to highlight the respective strengths and complementarities of each technology.

## Material & Methods

### Mouse model of liver metastases from colorectal cancer

All animal experimental procedures were approved by the animal ethics committee and received the authorization of the French Ministry of Higher Education and Research (ref: APAFIS#4937-2016041209236318 v7). HT29rvluc2 (human colorectal adenocarcinoma) cells stably expressing Luc2 firefly luciferase were obtained by using HIV virus particles for the transduction of the pGL4-Luc plasmid (Promega, France). Selection of stable transduced cells was performed by adding G418 (Invivogen, France) at 800 µg/mL to the culture medium and monoclonal cell populations were isolated by limiting dilution sub-cloning. HT29rvluc2 were then cultured in McCoy's medium supplemented with 10% heat-inactivated fetal bovine serum in a humidified atmosphere with 5% CO_2_. Twenty-four 6-week-old female NMRI nude mice (Janvier, France) were anesthetized (isoflurane/air 4% for induction and 1.5% thereafter) before an incision was made on the left flank and the spleen was pushed out of the abdomen. HT29rvluc2 cells (3×10^6^ cells in 20 µL 50/50 1X-PBS/Matrigel (Corning, USA)) were then implanted into the spleen. After a few seconds, once the Matrigel became solid, the spleen was replaced in the peritoneal cavity, and the peritoneum and skin were sutured. Two weeks later, the primary-tumor-bearing spleen was surgically removed following the same surgical method.

### *In vivo* bioluminescence imaging

Primary tumor growth and metastasis development were monitored weekly by *in vivo* bioluminescence imaging (BLI) for 6 weeks after tumor cell implantation. Five minutes before imaging, awake mice received an intraperitoneal injection of 150 µg/g of D-luciferin (Promega, France),were then anesthetized (isoflurane 4% for induction and 1.5% thereafter) and placed in the BLI system (IVIS Kinetic, PerkinElmer, USA), first on the left side and then in supine position. Semiquantitative data were obtained from bioluminescence images by drawing regions of interest on the liver area. Based on the bioluminescence signal from the liver, mice were distributed into two groups: early stage (n=12): BLI signal < 7.7×10^6^ ph/s; advanced stage (n = 12): BLI signal > 2.4×10^7^ ph/s.

### Contrast agent preparation

ICG (Infracyanine, SERB, France) and Angiostamp800 (Fluoptics, France) were diluted in PBS to concentrations of 200 and 50 µM, respectively. The contrast agents were injected intravenously via the tail vein (200 µL). For microCT contrast enhancement in the liver, Exitron Nano6000 (Myltenyi Biotec Gmbh, Germany) was injected intravenously (100 µL; 640 mg iodine/kg) the day before imaging.

### Contrast agent fluorescence and photoacoustic detectability

The dilutions of each contrast agent were prepared in PBS. Ten microliters drops of contrast agent at each concentration (n = 3) and 10-µL drops of PBS were then placed on a piece of parafilm, and fluorescence imaging was performed using the Fluobeam800 (Fluoptics, France) (laser excitation: 780 nm; emission: 820 nm long-pass filter). The fluorescence signal was quantified using Wasabi software (Hamamatsu photonics, Japan) and expressed as Relative Light Units normalized by exposure time (RLU/ms). Multispectral PAI was performed with the VevoLAZR (Fujifilm, Visualsonics Inc., Canada) to characterize the ICG and Angiostamp800 photoacoustic spectra. Samples of various contrast agent concentrations (n = 3) were placed into a 1 mm^3^ silicon cassette and submitted to spectroscopic PAI from 680 to 970 nm with a 1 nm step. Quantifications were performed using the manufacturer's software (VevoLab 3.1.1, Visualsonics Inc., Canada) and are expressed as PA values in arbitrary units (A.U.). For both imaging methods, the signal-to-noise ratio (SNR) was calculated for each concentration (contrast agent versus PBS), and the sensitivity threshold (SThr) was defined by the concentration at which the SNR reached 1.5.

### ICG blood pharmacokinetics

The ICG blood pharmacokinetics was acquired by real-time noninvasive PAI using the VevoLAZR system. Anesthetized mice (isoflurane/air: 2%) were placed on a heated plate, and the LZ250 transducer (256-element linear array; 21 MHz center frequency {13-24 MHz bandwidth}; 75 µm axial resolution; 150 µm lateral resolution; 25×25 mm^2^ image size) was positioned on the longitudinal axis of the carotid artery. The probe positioning was validated using the color Doppler mode. A catheter was placed into the tail vein, and photoacoustic image acquisition (800 nm) was initiated at a 5 images/second frequency. After 2 minutes of background acquisition, ICG was intravenously injected (200 µL; 200 µM), and its elimination from the blood was monitored for 30 minutes after injection. This procedure was performed on 3 healthy and 12 liver-metastasis-bearing mice. Nine other mice (3 healthy and 6 liver-metastasis-bearing mice) were injected intravenously (ICG: 200 µL; 200 µM), and blood samples (about 25 µL) were collected at various time points (1, 5, 10, 15, 20, 30, 45 minutes and 1 hour) by making a small incision at the end of the tail. Plasma was isolated by centrifugation (7200 g for 8 minutes) and 10 µL samples were then imaged by 2D fluorescence using Fluobeam800. For both monitoring methods, the ICG blood half-life was calculated (nonlinear regression) using GraphPad Prism version 6 (GraphPad, USA).

### Noninvasive PAI of the liver

3D ultrasound imaging and PAI were performed on the liver area using the VevoLAZR system. By using the “oxy-hemo” mode (750 and 850 nm), the total hemoglobin content (HbT) and tissue oxygen saturation rate (StO_2_%) were calculated according to Wang et al. 9. Then, 1, 24 and 48 hours after ICG injection and 24 hours after Angiostamp800 injection, whole-body 2D fluorescence imaging was performed before 3D PAI (at 800 nm) was carried out on the whole liver and 2D multispectral PAI (from 680 to 970 nm, 5 nm step) was implemented on a metastasis-bearing tissue slice. Spectral unmixing analyses were performed using VevoLab software according to 11.

### Combined fDOT and microCT imaging

One hour after ICG injection or 24 hours after Angiostamp800 injection, anesthetized mice (isoflurane/air: 2%) were placed in a homemade mobile animal holder for bimodal microCT/fDOT imaging. Three-dimensional fluorescence imaging was performed on the liver using the previously described fDOT system 3. MicroCT imaging was performed in line using the vivaCT40 system (Scanco Medical AG, Switzerland), whose acquisition parameters were 55 keV energy, 177 µA intensity, 300 ms integration time and 80 µm voxel isotropic resolution. MicroCT- and 3D-fluorescence-reconstructed volumes were merged using ImageJ software. The total fluorescence signal in the volume of interest (liver) was quantified and expressed as RLU.

### *Ex vivo* fluorescence imaging and histology

One hour after ICG injection or 24 hours after Angiostamp800 injection, mice were euthanized via cervical dislocation, and their livers were isolated. *Ex vivo* 2D fluorescence imaging was performed using the Fluobeam800 system before the livers were frozen in OCT Tissue-Tek (Sakura Finetek Europe B.V., Netherlands) and cut into 7-µm-thick slices using a cryostat. Slices were then stained with hematoxylin and eosin. In order to evaluate the presence of neo-formed vessels an immunological labelling of tissue sections was performed. Sequentially, a rat anti-mouse CD31 antibody and a HRP polymer anti-rat IgG (ImmPRESS™) were used for the enzymatic reaction therefore created with ImmPACT NovaRED kit. The presence of vessels was observed as a red-brown color and surrounding tissues were counterlabeled in violet-blue color. Slices were entirely scanned (mosaic) by an Axio Scan Z1 (Zeiss, Germany) under 20× magnification.

### Statistical analysis

Statistical analyses were performed using GraphPad Prism version 6 (GraphPad software, USA). Either one-way ANOVA or two-way ANOVA tests were used (*p-value<0.05; **p-value<0.01; ***p-value<0.001; ****p-value<0.0001).

## Results

### Mouse model of liver metastases from colon cancer

**Figure 1:**
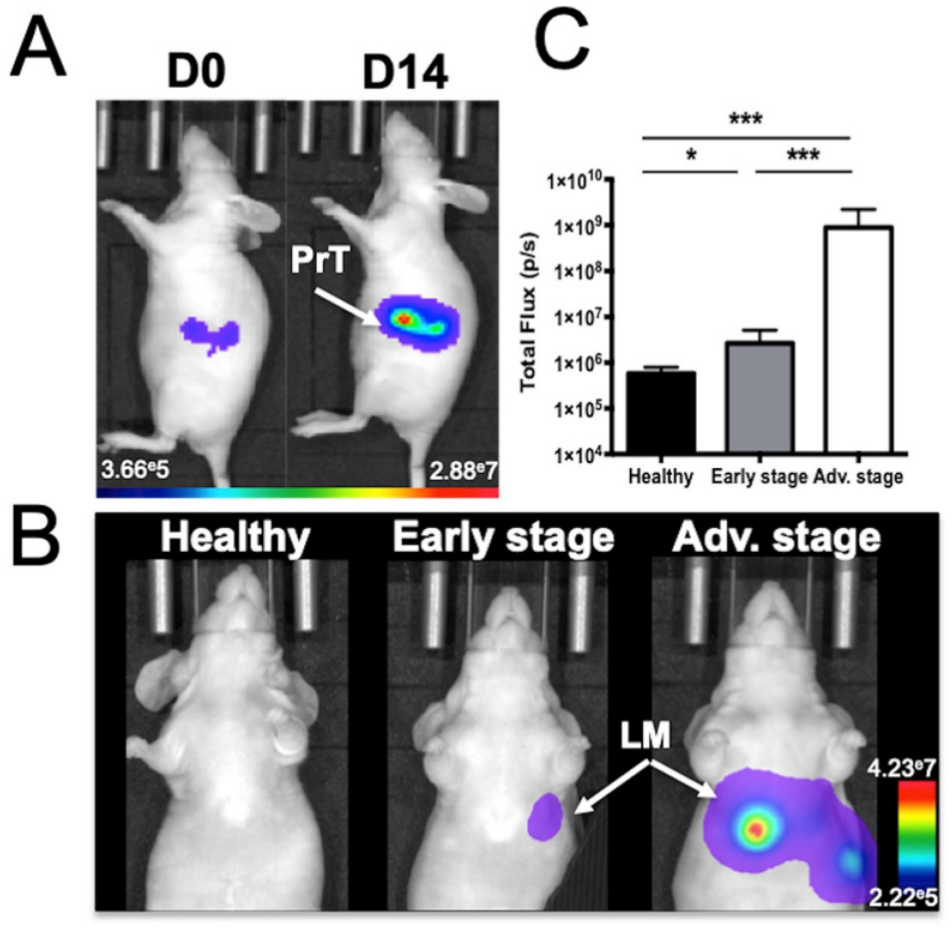
** Mouse model of liver metastases from colon cancer as monitored by *in vivo* bioluminescence imaging. A)** Imaging just after colon cancer cell implantation into the spleen (J0) and just before spleen resection at D14 (PrT: primary tumor) or **B)** at various stages of liver metastasis (LM) development. **C)** Abdominal bioluminescence quantification of mice in the early or advanced stage of LM development. n= 12 mice per group; statistical analyses: one-way ANOVA tests were used (*p-value<0.05; **p-value<0.01; ***p-value<0.001; ****p-value<0.0001).

**Figure 2:**
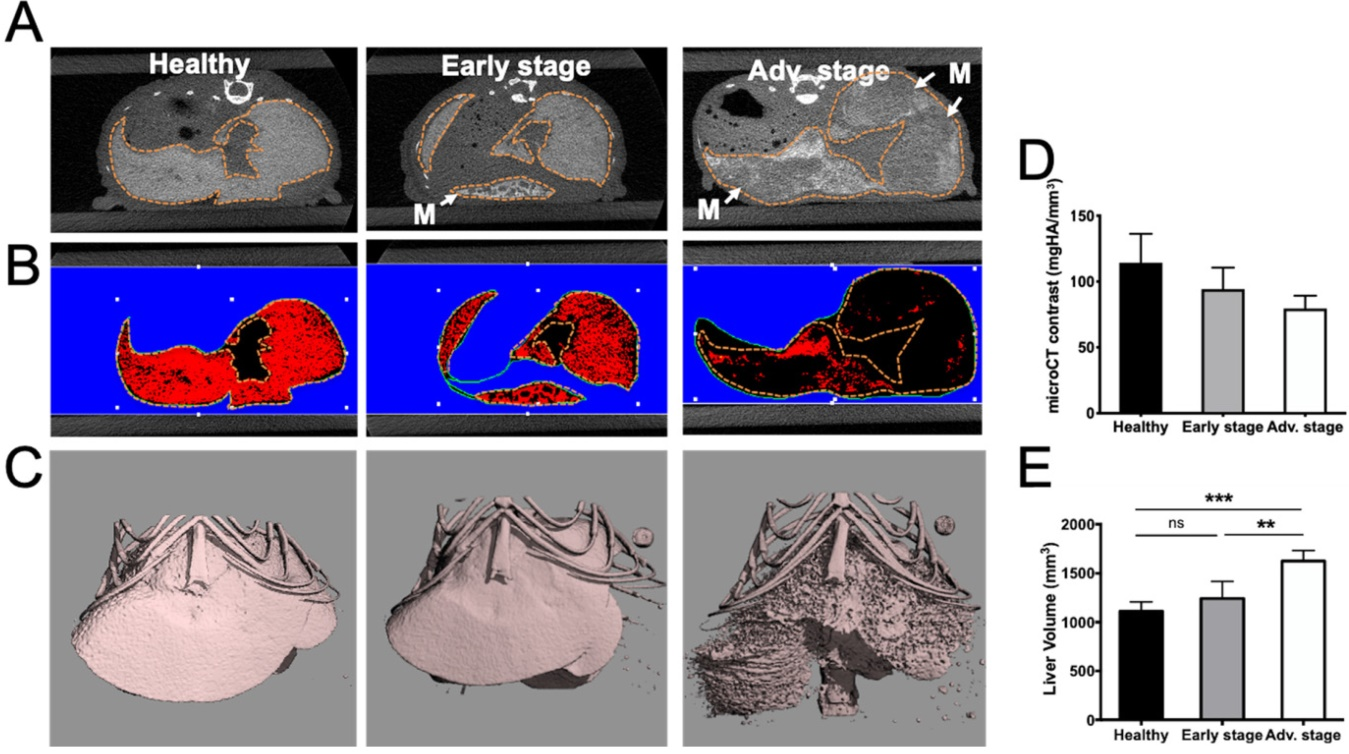
** Alteration of liver uptake at various stages of liver metastasis development as observed by microCT imaging after intravenous injection of alkaline earth-metal-based nanoparticles (Exitron Nano6000). A)** Transversal section of the liver. Doted lines indicate the liver outline. Tumors are flagged by white “M” and arrows. **B)** Color-coded contrast-based segmentation highlighting contrast agent uptake in healthy tissues (red). **C)** 3D contrast-agent-based segmentation of the liver. **D)** 3D quantification of liver contrast reporting the proportion of healthy tissue within the whole liver. **E)** Liver volume measured from contrast-based segmentation. n= 3 mice (healthy group), n= 6 mice per group (Early and Advanced); statistical analyses: one-way ANOVA tests were used (*p-value<0.05; **p-value<0.01; ***p-value<0.001; ****p-value<0.0001).

Human colorectal adenocarcinoma cells were surgically implanted into the spleen, which led to liver metastasis development after primary tumor resection (Figure 1). *In vivo* BLI was used to monitor the quality of the tumor cell implantation as well as primary tumor growth (Figure 1A) and abdominal metastasis development (Figure 1B). Quantification of the abdominal bioluminescence signal allowed for the rational distribution of the mice into two groups corresponding to early and advanced metastases stages (Figure 1C). Liver metastases were also detectable by *in vivo* microCT imaging using alkaline earth-metal-based nanoparticles for liver contrast enhancement (Figure 2A). Contrast-based segmentation showed that mice at the pathological advanced stage displayed altered liver uptake of nanoparticles as illustrated by the “nibbled” look of their liver compared to healthy mice (Figure 2C).

Exitron Nano6000-contrast was quantified in the whole liver and the decrease that was observed in pathological livers provided an estimation of the proportion of liver metastases (17.6±1.5% and 30.4±0.9%) relative to healthy tissue at early and advanced stages, respectively (Figure 2B, D).

In addition, whole liver volume measured after contrast-based segmentation was shown to be 50% larger in advanced stage mice compared to the healthy group (Figure 2E).

**Figure 3:**
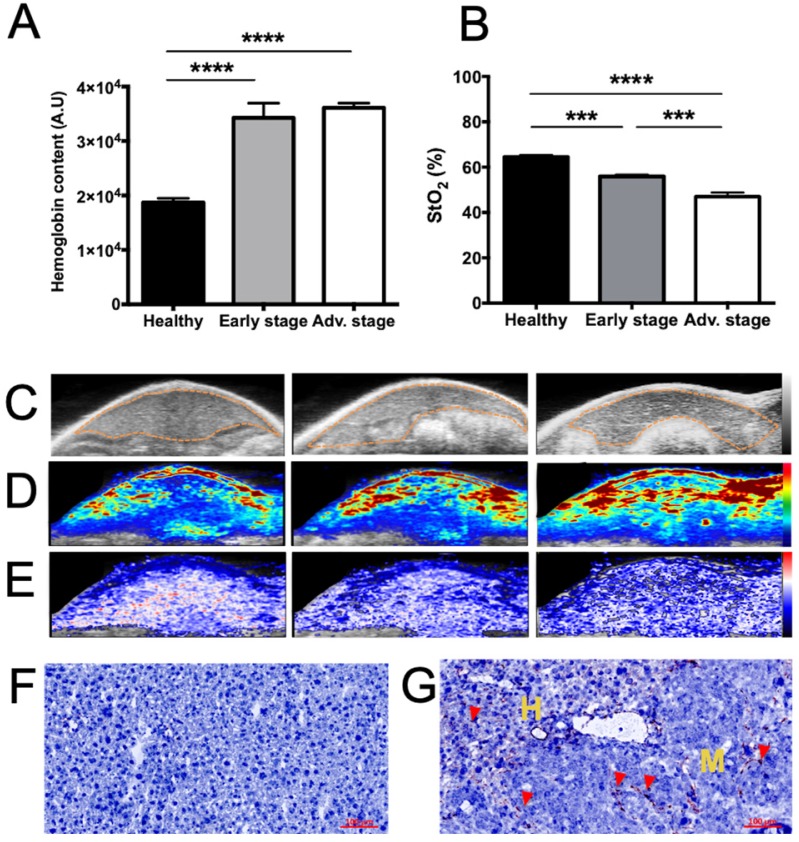
** Tumor-related angiogenesis and hypoxia at various stages of liver metastasis development as observed via contrast-agent-free 3D photoacoustic measurements.** 3D analyses of endogenous photoacoustic signals of the whole liver using the oxy-hemo protocol (750 and 850 nm wavelengths) for the monitoring of A) total hemoglobin content (HbT) and B) average tissue oxygen saturation (StO_2_). Liver sections from 3D ultrasound C) and photoacoustic imaging of D) HbT and E) StO_2_. The dotted line represents the ROI used for photoacoustic signal quantifications. n= 3 mice (healthy group), n= 6 mice per group (Early and Advanced); statistical analyses: one-way ANOVA tests were used (*p-value<0.05; **p-value<0.01; ***p-value<0.001; ****p-value<0.0001). Close-up look on F) healthy and G) metastasized tissues after CD31 labelling. Positive coloration is indicated by red arrows. Yellow “H” and “M” mentions refers to healthy and metastatic tissues respectively.

Using a 21 MHz ultrasound transducer and 750/850 nm laser light the whole liver was scanned and we were not able to identify individual metastases neither by US (Figure 3C) nor by contrast-agent-free PAI. In contrast, when analyzing the whole liver volume, mean HbT and StO_2_ were modified (Figure 3). HbT was shown to have slightly increased in the metastasis-bearing livers (Figure 3A, D), which may be related to tumor angiogenesis, while StO_2_ was shown to decrease gradually with metastasis development (Figure 3B, E), illustrating the tumor-related induction of hypoxia. In agreement with the HbT increase, CD31 immuno-staining showed that, in comparison with healthy tissues (Figure 3F), neo-vessels were formed in the liver metastases microenvironment (Figure 3G).

### Contrast agent fluorescence and photoacoustic detectability

**Figure 4:**
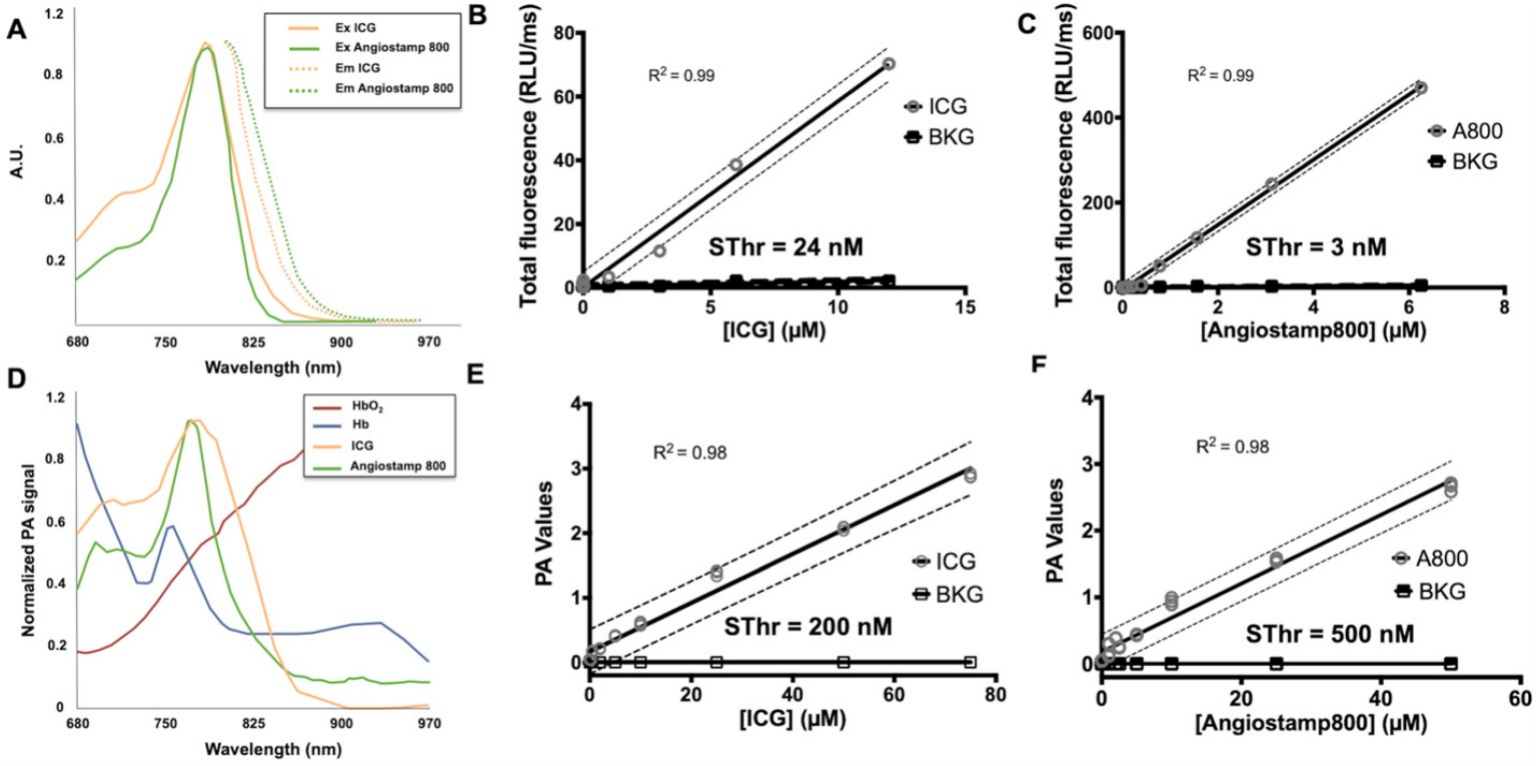
** Detectability of indocyanine green (ICG) and Angiostamp800 (A800) by fluorescence and photoacoustic imaging. A)** Absorption and fluorescence spectra of ICG (orange) and Angiostamp800 (green).** B)** Fluorescence signal of various concentrations (n=3 samples/concentration) of ICG, **C)** Angiostamp800 and fluorescence background noise (BKG) measured by the Fluobeam®800 (excitation 780 nm; emission > 830 nm). The sensitivity threshold (SThr) corresponds to the concentration that provides a signal-to-noise ratio (contrast agent versus PBS) of 1.5.** D)** Photoacoustic spectra of ICG (orange), Angiostamp800 (green), oxyhemoglobin (dark red) and deoxyhemoglobin (dark blue). **E)** Photoacoustic signal of various concentrations (n=3 samples/concentration) of ICG, **F)** Angiostamp800 and photoacoustic background noise (BKG) measured by spectral unmixing analyses from spectroscopic acquisition (680-970 nm). The sensitivity threshold (SThr) corresponds to the concentration that provides a signal-to-noise ratio (contrast agent versus PBS) of 1.5.

ICG and Angiostamp800 displayed specific absorption and fluorescence spectra (Figure 4A) in the near-infrared region fully compatible with their detection by Fluobeam™800 (Figure 4B, C), which provided linear detections from 10 µM and below (r^2^ = 0.99) and very low sensitivity thresholds (Sthr: 24 and 3 nM, respectively). Their photoacoustic profiles were very similar to their absorption spectra (Figure 4D), and their measurements in sample cassettes were highly repeatable (coefficients of variation between 0.5 and 6.9 %). Signal quantifications were linear from 50 µM and below (r^2^ = 0.98), providing photoacoustic sensitivity thresholds of 200 and 500 nM, respectively (Figure 4E, F). Interestingly, these contrast agents displayed photoacoustic spectra clearly distinguishable from those of oxy- and deoxy-hemoglobin (Figure 4D), which indicates the possibility of spectral unmixing.

### ICG blood pharmacokinetics

**Figure 5:**
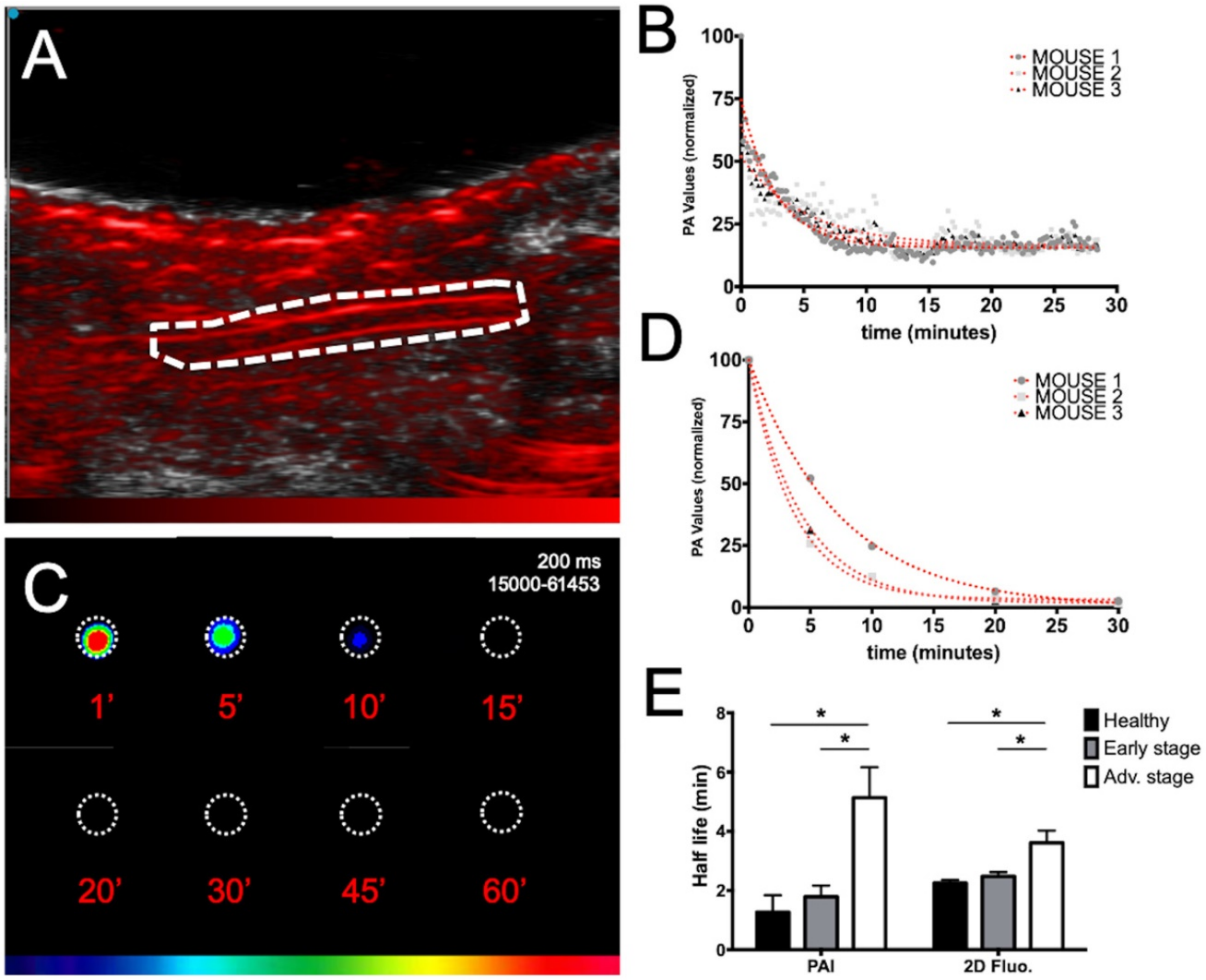
** Evaluation of ICG blood pharmacokinetics by real-time noninvasive photoacoustic imaging and *ex vivo* fluorescence imaging of plasma samples.** A) Photoacoustic imaging of the carotid artery (dotted line; longitudinal section) before ICG injection. B) Real-time *in vivo* monitoring of ICG photoacoustic signal (800 nm) from the carotid artery of three healthy mice after intravenous injection of ICG and non-linear fits of data. C) *Ex vivo* fluorescence imaging of plasma samples collected at different time points after intravenous injection of ICG. D) Non-linear fits of *ex vivo* fluorescence signal of plasma samples from three healthy mice after intravenous injection of ICG. E) ICG blood half-lives calculated either from real-time *in vivo* monitoring of ICG photoacoustic signal from the carotid artery or from *ex vivo* fluorescence signal of plasma samples collected from mice at various stages of liver metastasis development. n= 3 mice (healthy group), n= 6 mice per group (Early and Advanced); statistical analyses: non-linear fitting curves (one-phase decay) and two-way ANOVA tests were used (*p-value<0.05; **p-value<0.01; ***p-value<0.001; ****p-value<0.0001).

The ICG blood pharmacokinetics were evaluated in mice at various stages of liver metastasis development by both noninvasive real time PAI on the carotid artery (Figure 5A, B) and *ex vivo* fluorescence imaging of plasma samples collected at various time points (Figure 5C, D).

Individual blood half-lives were calculated from nonlinear regression (Figure 5B, D) of each mouse PAI or fluorescence data and averaged half-lives were found to be 1.3±1.0 minutes for PAI and 2.3±0.2 minutes for fluorescence imaging in healthy mice (Figure 5E). A marked increase in the circulation time was observed for the mice at advanced stages compared to the circulation time of the mice at early stages and healthy mice regardless of the imaging modality. Interestingly, although slightly higher variability was observed in the noninvasive photoacoustic measurements, the gathered information was the same as that obtained from *ex vivo* fluorescence detection on collected samples.

### ICG liver kinetics

**Figure 6:**
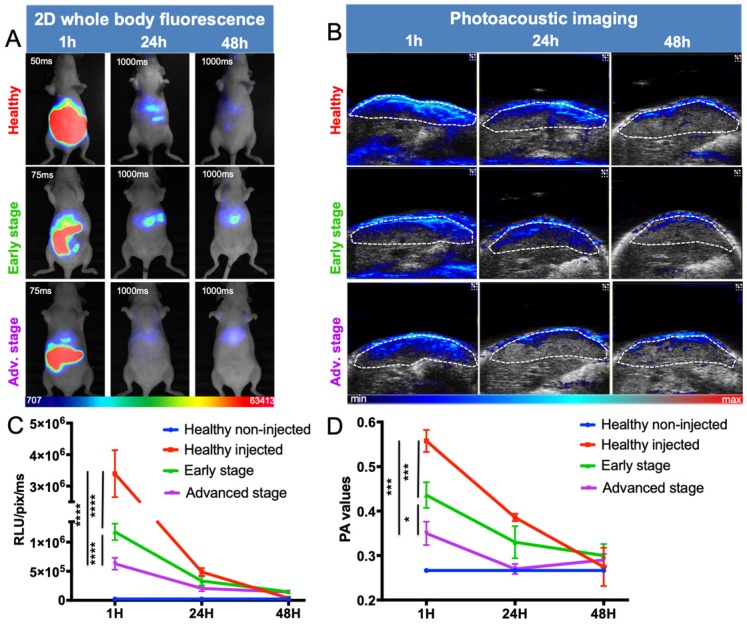
***In vivo* distribution kinetics of ICG at various stages of liver metastasis development by fluorescence and photoacoustic imaging.** A) 2D whole-body fluorescence imaging 1h, 24h and 48h after intravenous injection of ICG at various stages of liver metastasis development. Fluorescence signals being highly different between early and late time points, the images were taken with optimized exposure time. B) Liver sections from 3D ultrasound and photoacoustic imaging (800 nm). The dotted white line represents the ROI used for photoacoustic signal quantifications. C) Kinetics of fluorescence and D) photoacoustic signals from the liver. n= 3 mice (healthy group), n= 6 mice per group (Early and Advanced); statistical analyses: two-way ANOVA tests were used (*p-value<0.05; **p-value<0.01; ***p-value<0.001; ****p-value<0.0001).

Following intravenous injection of ICG, healthy mice displayed a strong signal in the liver, decreasing to background noise 48 hours after injection (Figure 6). Liver-metastasis-bearing mice followed the same elimination profile, but their early liver uptake was significantly impaired, as demonstrated by both noninvasive whole-body 2D fluorescence and 3D PAI (Figure 6A, B). More precisely, when looking at ICG fluorescence or photoacoustic signals in the liver 1 hour after injection, the more advanced the development of metastases, the lower the liver signal (Figure 6C and D).

### Monitoring of liver metastasis development by noninvasive 3D multimodal imaging with ICG and Angiostamp800

**Figure 7:**
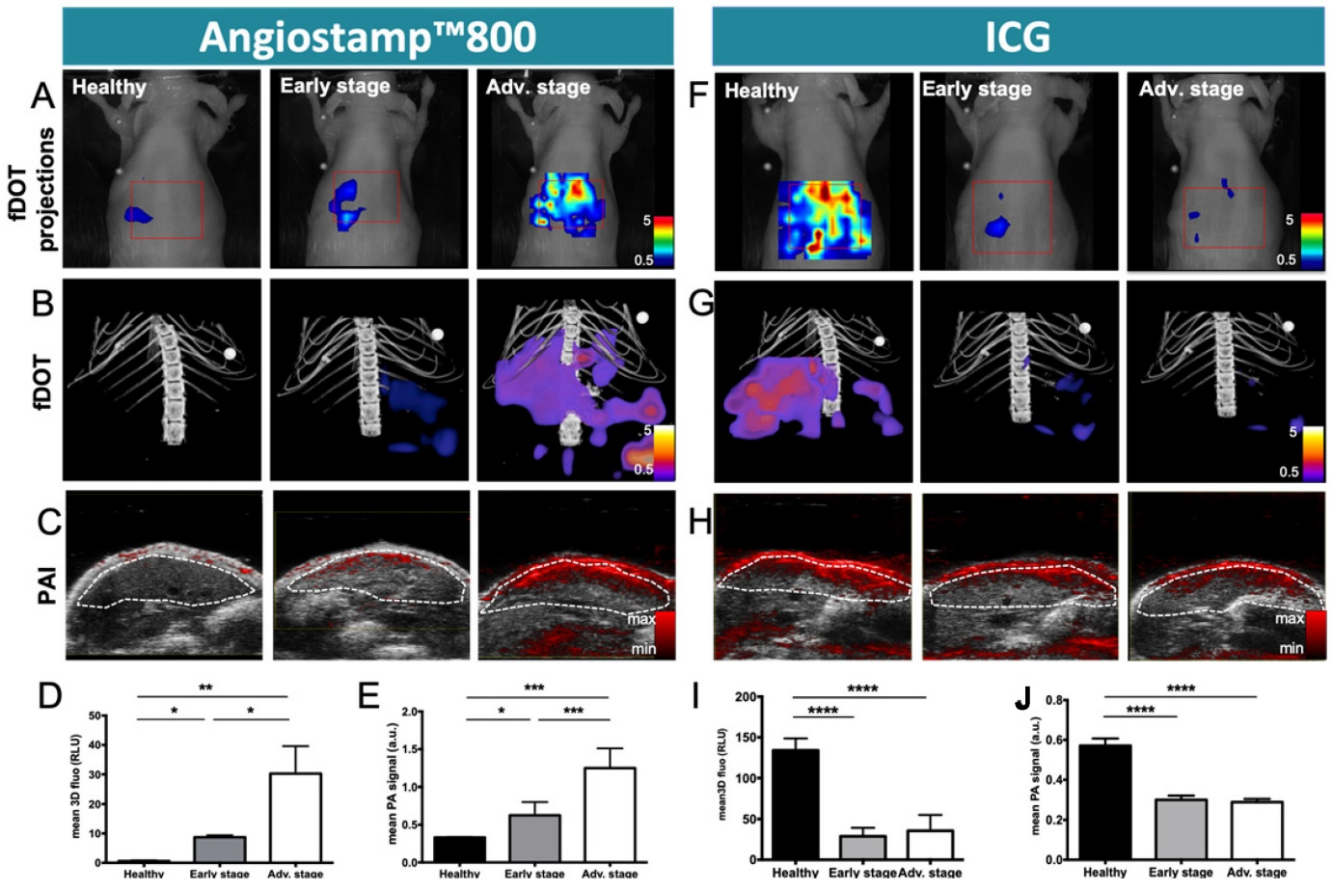
** Monitoring of liver metastasis development by noninvasive 3D fluorescence and photoacoustic imaging with Angiostamp800 and ICG.** Fluorescence and photoacoustic imaging were performed 1 hour after ICG intravenous injection or 24 hours after Angiostamp800 intravenous injection. A) Projections from 3D fluorescence imaging of the liver with Angiostamp800. B) 3D bimodal fluorescence/microCT imaging of the liver with Angiostamp800. C) Liver sections from 3D ultrasound and photoacoustic (800 nm) imaging with Angiostamp800. The dotted white line represents the ROI used for photoacoustic signal quantifications. D) Fluorescence and E) photoacoustic signals measured from the liver with Angiostamp800. F) Projections from 3D fluorescence imaging of the liver with ICG. G) 3D bimodal fluorescence/microCT imaging of the liver with ICG. H) Liver sections from 3D ultrasound and photoacoustic (800 nm) imaging with ICG. I) Fluorescence and J) photoacoustic signals measured from the liver with ICG. n= 3 mice (healthy group), n= 6 mice per group (Early and Advanced); statistical analyses: one-way ANOVA tests were used (*p-value<0.05; **p-value<0.01; ***p-value<0.001; ****p-value<0.0001).

**Figure 8:**
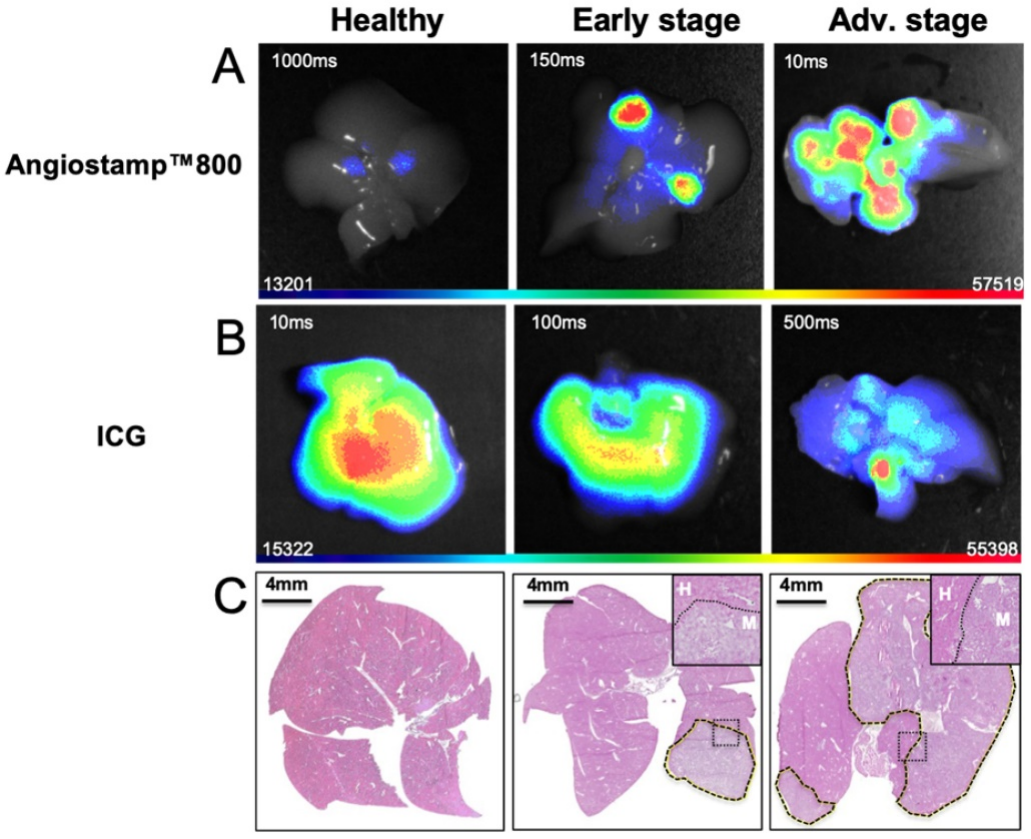
***Ex vivo* 2D fluorescence of isolated liver at various stages of liver metastasis development.** Twenty-four hours after intravenous Angiostamp800 injection or B) one hour after intravenous ICG injection. C) Hematoxylin and eosin staining of liver slices (liver metastases delineated by dotted lines) with inserted close-up look on frontier region between metastasis (M) and Healthy tissue (H).

Based on ICG liver kinetics, the time point 1 hour post injection was chosen for liver metastases exploration at various developmental stages while Angiostamp800 imaging was performed 24 hours injection following the manufacturer recommendation for optimal nonspecific signal wash out (Figure 7). Using Angiostamp800, liver-metastasis-bearing mice displayed liver signals that were clearly detectable by both fDOT and PAI (Figure 7A-C). From the fluorescence measurements, mice at advanced stages presented a significantly higher signal (30.25±18.70 RLU) than mice at early stages (8.71±1.55 RLU); while healthy mice showed only a very weak signal (0.58±0.44 RLU) (Figure 7D). These observations were confirmed by *ex vivo* fluorescence imaging on the isolated livers (Figure 8B). *In vivo* 3D photoacoustic measurements provided similar patterns (Figure 7C); the more advanced the metastasis development was, the higher the signal was (×1.9 for early stage and ×3.8 for advanced stage, compared to the signal in healthy mice), thus allowing precise discrimination between all stages (Figure 7D). Conversely, using ICG, the livers of the healthy mice displayed high fluorescence (Figures 7A,B & 8A) and photoacoustic signals (Figure 7C), and these signals were significantly decreased (p<0.0001) in liver-metastasis-bearing mice (-80.7 to -88.1 % for fluorescence imaging (Figure 7I) and -47.3 to -49.3 % for photoacoustic imaging (Figure 7J) compared to the signals of the healthy mice). However, for both imaging modalities, there was no significant difference in liver signal between the early and advanced stages (Figure 7I, J).

### *In vivo* multispectral PAI

**Figure 9:**
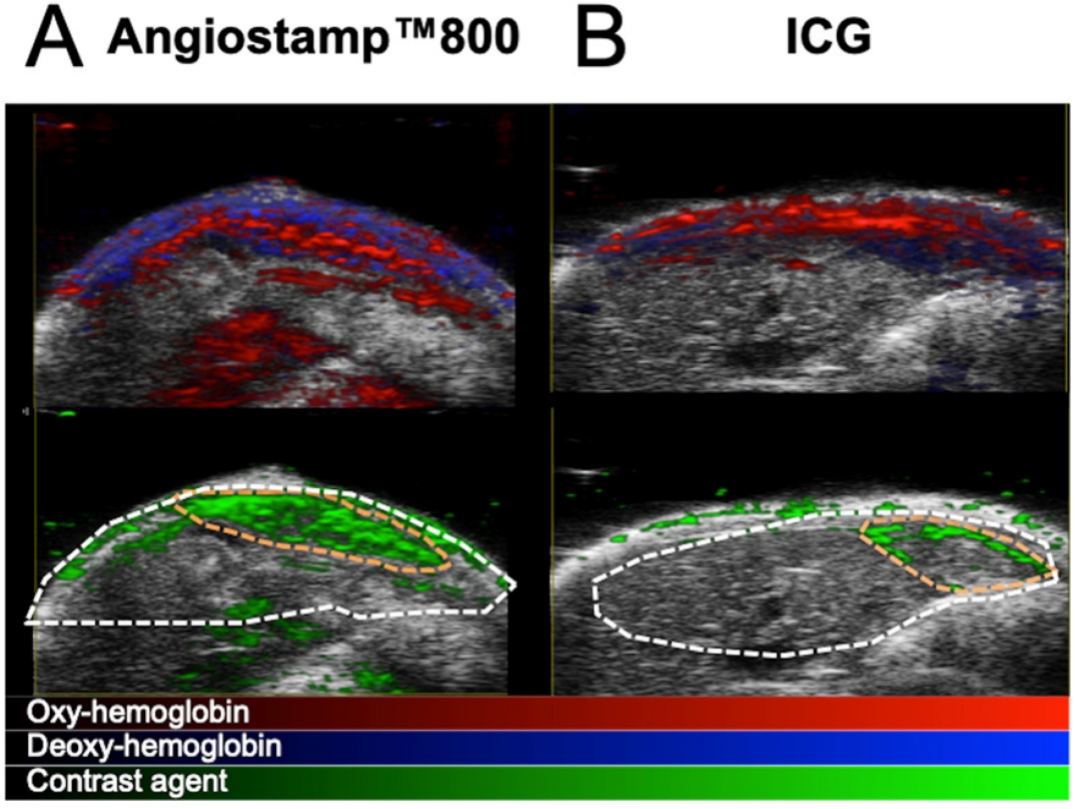
***In vivo* mapping of oxyhemoglobin, deoxyhemoglobin and tracer distribution by multispectral photoacoustic imaging.** Liver sections from 3D ultrasound and multispectral photoacoustic imaging with Angiostamp800 (A) or ICG (B) injected 24 hours or 1 hour before imaging, respectively (red: oxyhemoglobin, blue: deoxyhemoglobin, green: Angiostamp800 or ICG).

Having previously defined the specific photoacoustic spectra of the contrast agents (Figure 4D), multispectral photoacoustic acquisitions were performed on liver-metastasis-bearing mice after injection of ICG or Angiostamp800 (Figure 9). Spectral unmixing analysis could be implemented and provided simultaneous monitoring and visualization of HbT, StO_2_ and contrast agent concentration within the liver (Figure 9). Using Angiostamp800, a contrast-agent-specific photoacoustic signal and increased HbT were observed at a metastasis site (Figure 9A). However, ICG provided low, but ring-shaped, specific photoacoustic signals together with high HbT at a metastasis site (Figure 9B).

## Discussion

In this study, we precisely characterized the development of liver metastases from human colon cancer in a murine model 12 by *in vivo* multimodal imaging using bioluminescence, microCT, fluorescence imaging and multispectral PAI.

The use of several imaging modalities thus offered combined and cross-validated information. First, in a conventional way, the metastatic invasion could be followed and graded using bioluminescence imaging of the tumor cells thanks to their luciferase expression. Then, microCT using nanoparticles for liver contrast enhancement illustrated that mice at the pathological advanced stage displayed altered liver uptake compared to healthy mice. This indirect tumor evaluation method is based on the elimination function of the Kupffer cells in the liver which is impaired by the presence of tumors. However, the sensitivity of this approach was quite low since mice at the early stage did not appear significantly different from those in the healthy group. Additionally, this procedure required the injection of alkaline earth-metal-based nanoparticles whose potential toxicity precludes repeated injections for tumor growth longitudinal follow-up 13.

Conversely, contrast-agent-free PAI was used to detect the respective amounts of oxy- and deoxyhemoglobin, and HbT was shown to have inconsistently increased in metastasis-bearing livers, which may be related tumor angiogenesis, while StO_2_ was shown to decrease gradually with metastasis development, illustrating the induction of hypoxia.

Liver metastases exploration by molecular imaging was then performed, and the ICG and Angiostamp800 contrast agents were characterized for their use in both fluorescence imaging and PAI. These two contrast agents displayed specific absorption and fluorescence spectra in the near infrared region, where autofluorescence, light absorption and scattering through tissue are moderated 14. Following light excitation, their fluorescence quantum yield was moderate (12-14 %), and part of the absorbed energy was converted into heat and generated a photoacoustic effect. Overall, both fluorescence and photoacoustic signals of these two contrast agents were shown to be linear in wide concentration ranges, thus allowing quantitative signal analysis. Beside remarkable sensitivity thresholds for fluorescence (a few nanomolar) PAI displayed substantial sensitivity thresholds at a few hundred nanomolar, which offers wide detection potential.

These contrast agents' dual behavior presents very interesting prospects for associating the two molecular imaging modalities and thus combining their respective strengths. On one hand, fluorescence is incomparably sensitive, but both resolution and signal quantification are impacted by tissue depth because of light absorption and scattering by the tissues 15. Fluorescence diffuse optical tomography attempts to circumvent this weakness by taking into account these two optical parameters for signal correction 2, however fluorescence detection is still limited to a few millimeters in depth; millimeter resolution being the best possible in any case. Moreover fluorescence tomography requires multiple image acquisitions that impair temporal resolution and thus preclude real-time pharmacokinetic studies.

On the other hand, PAI resolution is driven mainly by ultrasound detection and is thus less affected by depth than fluorescence. Consequently, a 100 µm resolution can be achieved at 3 cm depth using a 9-18 MHz transducer and resolution can be even higher when using high frequencies to study tissues at a depth of less than 1.5 cm (40 µm axial resolution at 32-55 MHz). In terms of photoacoustic signal detection, one should be aware that the higher the resolution is the lower the sensitivity will be. In the present work a 21 MHz transducer was used to explore the whole liver volume and using this setting we were not able to identify individual metastases neither by US nor by contrast-agent-free PAI. Higher frequency such as 40 MHz would provide better spatial resolution that may allow single metastasis isolation, in particular if using contrast enhanced ultrasound imaging with dedicated microbubbles, but it would concomitantly provide less in-depth access and lower sensitivity for photoacoustic signal detection.

By using several wavelengths of laser light, multispectral PAI offers simultaneous monitoring of endogenous compounds and exogenous contrast agent signals, providing noninvasive mapping of specific tracers' *in vivo* distribution combined with functional data about angiogenesis and tissue oxygenation 11,16. Significant advantage is also offered by the use of a transducer which enables real-time repositioning of photoacoustic signal within anatomical information from ultrasound. Similar to 2D-fluorescence imaging, PAI can provide real-time imaging when monitoring one tissue slice and in the present study, we took advantage of this to perform the noninvasive, real-time monitoring of ICG blood pharmacokinetics and, therefore, determine its elimination half-life at various disease stages. This procedure was performed in parallel with fluorescence imaging on plasma samples collected at various time points, and the blood half-lives calculated from both imaging modalities in healthy mice were in accordance with each other and with the literature that describes the ICG blood half-life as approximately 2-4 minutes for healthy rodents 17. We also demonstrated by this technique that ICG half-life was modified according to the progression of the disease. Similarly, ICG has been used in patients for a very long time to assess liver function from blood samples. Our results demonstrated that ICG blood pharmacokinetic monitoring via noninvasive PAI could be used as an indirect marker of liver metastasis development; a marked increase in the circulation time was observed in mice at advanced stages compared to mice at early stages and to healthy mice. ICG being eliminated by the liver, its concentration in blood is directly affected by any liver impairment which is slowing down the process. Similar to contrast-agent-based microCT evaluation, this approach was not able to discriminate mice at an early disease stage from healthy mice. Evaluating the kinetics of ICG elimination thus provides information on the pathophysiological status of the liver, but it is not specific to cancer development.

Beside liver function evaluation, ICG has recently been applied to a new scope of application for intraoperative fluorescence-guided surgery 18. In addition to its specific catabolism by hepatocytes, this contrast agent can also be passively and non-specifically retained into tumors 6. ICG has been shown to facilitate tumor resection in several cancer types in humans 19-23 and in particular in the context of hepatic metastases from colorectal cancer 24-26.

We therefore evaluated the potential of ICG in the noninvasive exploration of liver metastases using both fluorescence imaging and PAI. Whole-body 2D-fluorescence imaging was used to characterize ICG biodistribution kinetics and in particular liver uptake, however, when using 2D-fluorescence imaging, distinguishing the liver signal from the gut signal was an issue, and only fDOT imaging combined with microCT allowed for precise delineation of liver volume and, thus, rigorous liver signal quantification. Using ICG, both fDOT and PAI allowed for the discrimination between liver-metastases-bearing mice and healthy ones, but there was no significant difference between early and advanced stages regardless of the imaging modality.

Miyata and colleagues 25 performed both intraoperative fluorescence imaging and PAI on surgical specimens from patients with hepatocellular carcinoma or colorectal liver metastasis that had been injected with ICG prior to hepatectomy and found consistent images between the two modalities. Interestingly, they observed that while ICG accumulated in hepatocellular carcinoma nodules, in the case of colorectal liver metastases ICG accumulated not in the cancerous tissue but rather in the peri-cancerous hepatic parenchyma as previously described in the context of fluorescence-guided surgery of liver metastases from colorectal carcinoma 24. In our study, spectral unmixing analysis of the ICG photoacoustic signal in liver metastasis revealed a “rim-type” signal, delineating a metastasis site.

Angiostamp is a tumor-targeting contrast agent that acts via the specific targeting of α_v_β_3_ integrin, which is overexpressed in angiogenesis and aggressive migrating cancer cells 27. Angiostamp has already been shown to provide a strong fluorescence signal in tumor tissue with a low nonspecific background, generating very good tumor contrast in various animal models, including models of peritoneal carcinomatosis 28-31, fibrosarcoma 32, osteosarcoma 33, head and neck squamous cell carcinoma 34 and bone metastasis from breast cancer origin 35.

In this study, Angiostamp800 was introduced for the first time as a new photoacoustic contrast agent whose tumor-targeting specificity and bimodal properties were used for the monitoring of liver metastases progression by combined PAI and fluorescence imaging.

Using Angiostamp800, liver metastases were clearly detectable by both fDOT and PAI. For both imaging modalities, the more advanced the metastasis development was, the higher the signal, thus allowing for precise discrimination between all disease stages and the healthy state in mice. Nevertheless it should be noted that PAI provided higher significance in the stages discrimination than fDOT. In the end, this result highlighted the significant improvement achieved by using a tumor-specific marker such as Angiostamp800 compared to ICG passive accumulation in the tumor by the EPR effect.

This study reported the first use of Angiostamp800 as a new photoacoustic organic contrast agent, whose tumor-targeting specificity and bimodal properties enabled the combined use of PAI and fluorescence imaging for the noninvasive monitoring of liver metastasis progression *in vivo*. This was the first time that these two molecular imaging modalities were closely associated and compared in a complete preclinical study with longitudinal follow-up, and this provided a better understanding of their respective strengths and complementarities. We thus presented multimodal imaging methods for noninvasive cancer development monitoring that represent powerful tools for the development and preclinical evaluation of new therapeutic strategies against cancer.

## Abbreviations

2D: two-dimensional
3D: three-dimensional
AU: arbitrary units
BLI: bioluminescence imaging
CO_2_: carbon dioxide
EPR effect: enhanced permeability and retention effect
fDOT: fluorescence diffuse optical tomography
HbT: hemoglobin content
ICG: indocyanin green
MicroCT: micro-computed tomography
PA: photoacoustic
PAI: photoacoustic imaging
PBS: phosphate-buffered saline
SNR: signal-to-noise ratio
STht: sensitivity threshold
StO_2_: tissue oxygen saturation
RLU: relative light units
